# An *In Vivo* Fluorescence Resonance Energy Transfer-Based Imaging Platform for Targeted Drug Discovery and Cancer Therapy

**DOI:** 10.3389/fbioe.2022.839078

**Published:** 2022-02-14

**Authors:** Fuqiang Xing, Nana Ai, Shigao Huang, Cheng Jiang, Muhammad Jameel Mughal, Wei Ge, Guanyu Wang, Chu-Xia Deng

**Affiliations:** ^1^ Cancer Center, Faculty of Health Sciences, University of Macau, Macau SAR, China; ^2^ Department of Biology, School of Life Sciences, Guangdong Provincial Key Laboratory of Computational Science and Material Design, Southern University of Science and Technology, Shenzhen, China; ^3^ Guangdong Provincial Key Laboratory of Biomedical Imaging and Guangdong Provincial Engineering Research Center of Molecular Imaging, The Fifth Affiliated Hospital, Sun Yat-sen University, Zhuhai, China; ^4^ Centre of Reproduction, Development and Aging, Faculty of Health Sciences, University of Macau, Macau SAR, China; ^5^ School of Life and Health Sciences, The Chinese University of Hong Kong, Shenzhen, China

**Keywords:** apoptosis, cancer therapy, drug discovery, FRET technique, xenograft model

## Abstract

In the present study, an efficient *in vivo* drug screening platform is established based on FRET technique. We transfected cancer cells with FRET-based caspase-3 (C3) sensor and validated the cell lines by detecting the change in FRET signal caused by the *in vitro* drug-induced cell apoptosis. Furthermore, the C3 expressing cancer cells were then injected into zebrafish embryos and nude mice to establish the corresponding *in vivo* xenograft models. We found that cancer cell lines expressing C3 were effective in detecting cell death following drug treatment, including the detection of the tipping point of apoptosis. The drug-induced cell apoptosis was also observed in both zebrafish embryos and nude mice xenograft models. Overall, the FRET-based platform, through *in vivo* imaging, is potentially useful to improve drug screening efficiency.

## Introduction

Personalized cancer treatment requires reliable prediction of molecular responses to chemotherapy in individual patients (Tiriac et al., 2018). Although multiple preclinical models have been widely used in anti-cancer drug screening and toxicity studies in the past decade (Bisht and Feldmann, 2019; Guillen et al., 2021), they have respective limitations in terms of efficiency and accuracy, and their interchangeability is poor (Lu et al., 2017; Verjans et al., 2018). Current animal models for drug screening are time-consuming, insufficient, cost-ineffective, and usually require expensive equipment (Fernandez et al., 2021). These limitations compromise the availability and reliability of the current models, resulting in unnecessary delays in critical drug approvals (Van Norman, 2019). Therefore, new approaches and improved preclinical models are required to improve our ability in precision cancer therapy, including the optimization of drug discovery and even the discovery of new treatment modalities (Kalimuthu et al., 2017; Du et al., 2018).

Recent advances in anti-cancer drug discovery mainly focus on the development of new models to test/screen drugs that cause rapid apoptosis of cancer cells (January 2019). Because caspase-3 activation is a hallmark of apoptosis, the visualization of caspase-3 activation has been employed to identify apoptosis by a number of reports (Luo et al., 2001; Takemoto et al., 2003). In a previous study, HeLa cells employing Förster resonance energy transfer (FRET) technique was used to detect caspase-3 activation, which affected energy transfer between the cyan fluorescent protein (CFP) and yellow fluorescent protein (YFP) and lowered the FRET ratio of the probe (Luo et al., 2001). In our earlier study, we also used FRET to visualize the dynamics of doxorubicin (DOX) treated single cells and test the pharmacodynamics of drug treated tumor slices in a 3D-tumor slice culture (3D-TSCs) model (Xing et al., 2021). However, whether the FRET technique can be applied to animal models and drug evaluation remains yet to be explored. Since a whole living organism is vital to uncover the characteristics of cancer metastasis and progression, it is thus imperative to develop quick *in vivo* drug testing method in animal experiments.

**FIGURE 1:**
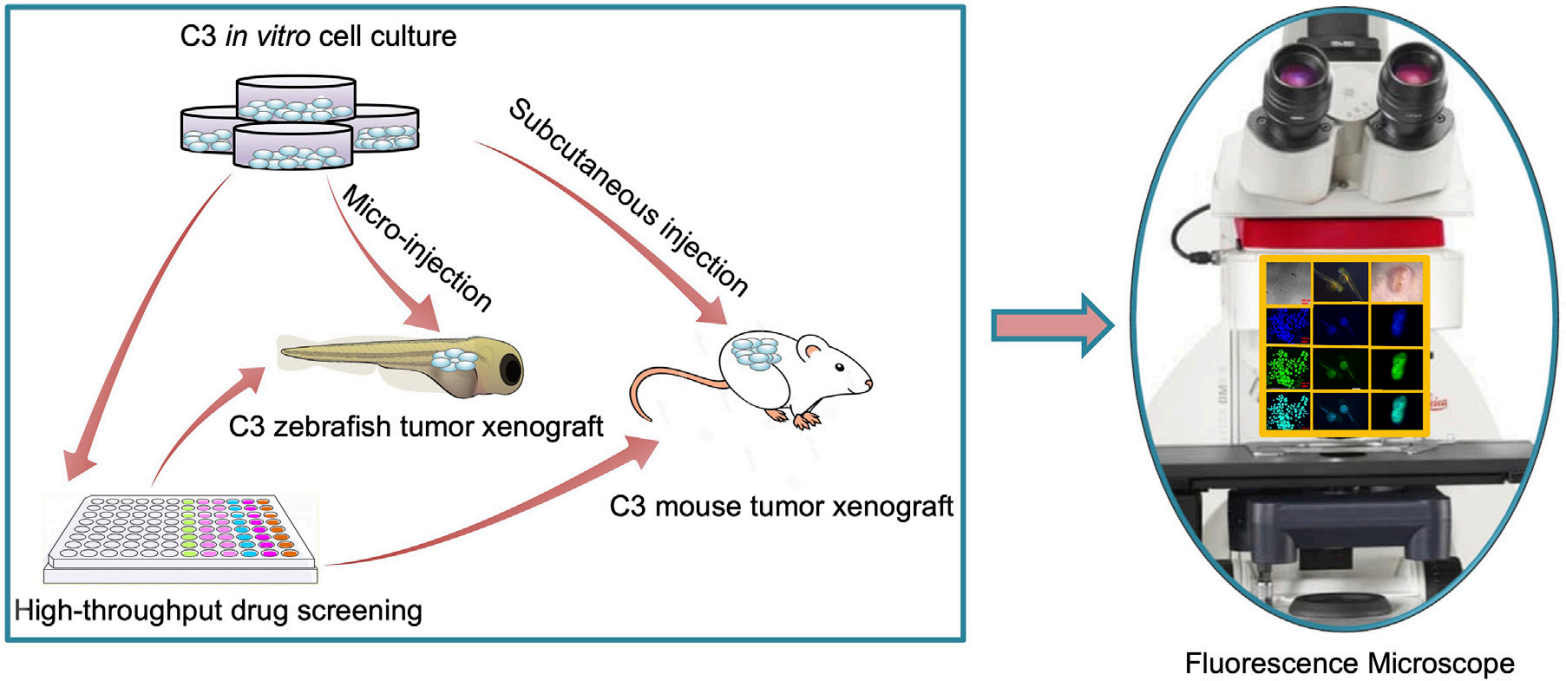
Schematic of FRET-based xenograft model for rapid drug discovery.

In the present study, we developed an *in vivo* FRET-based anti-tumor drug evaluation platform in xenograft zebrafish and nude mouse (Figure 1), and examined the proficiency of the platform in visualizing apoptosis dynamics and evaluating the efficacy of chemotherapeutic drugs.

## Material and Methods

### Cell Lines and Drugs

The human breast cancer cell lines (MDA-MB-231), the human liver cancer cell lines (HepG2), and the human lung cancer cell line (A549) were obtained from American Type Culture Collection and were cultured in a humidified incubator at 37°C, 5% CO_2_, and 95% air with Dulbecco’s modified Eagle’s medium (DMEM) supplemented with 10% fetal bovine serum (FBS) and 1% antibiotics (penicillin and streptomycin, Life Technologies). The drugs were purchased from Selleck Chemicals (Houston, TX, United States) and Sigma-Aldrich (Shanghai, China). All the drugs were prepared at a concentration of 10 mM and then diluted with culture medium to obtain their working concentrations.

### DNA Transfection

pSensor C3 was a generous gift from Prof. Kathy Qian Luo at the University of Macau. Plasmids were purified using NucleoBond® Xtra kit and linearized with restriction endonuclease *StuI*. After linearization, cancer cells (MDA-MB-231-C3, HepG2-C3, and A549-C3) were transiently transfected with plasmid DNA using Lipofectamine LTX with Plus reagent (Invitrogen) according to the manufacturer’s instructions. Transfection of adherent cells were performed using an optimized transfection protocol. The cells at concentrations of 0.5-2 × 10^5^/ml were seeded into a 12-well culture plate with 500 µL of antibiotics-free growth medium per well. Reagent complexes for transfection were prepared according to the manufacturer’s instructions. Reaction solutions were incubated for 20 min to form the complexes. The cells were incubated at 37°C in a CO_2_ incubator for 6 h, after which the medium was replaced with the normal medium. The C3 labeled cells were sorted by using a flow cytometer (FACS Aria III, BD Biosciences) to purify the stable cells.

### Time-Lapse Imaging of Cells and Tumors

For time-lapse imaging of cancer cells, the images of live cancer cells expressing C3 were captured using a Nikon Eclipse Ti-E inverted microscope and were then analyzed with NIS Elements AR software (Nikon). Nikon Eclipse Ti-E fluorescent microscope was equipped with a charged couple device (CCD) camera (ORCA R2, Hamamatsu Photonics KK, Hamamatsu, Japan) and CO_2_ incubation chamber (Matsunami Glass Ind., Ltd., Osaka, Japan). During the detection of YFP (525 nm) and CFP (480 nm) signals, the temperature of the chamber was kept at 37°C in an atmosphere of 5% CO_2_. In the merged FRET images, live cells appeared in cyan while apoptotic cells appeared in blue. The YFP and CFP images were analyzed to calculate the number of apoptotic cells and total cells using R Studio software. YFP signal of cells in the YFP images was counted and considered as total cells. The percentage of apoptotic cells in the images was also counted using the following formula: apoptotic cells (%) = blue cells in the merged images/total cells in the YFP images (%).

### Zebrafish Embryo Xenograft Model

The zebrafish embryo xenograft model was generated as previously reported (Nicoli and Presta, 2007). Briefly, zebrafish embryos at 2 dpf were arranged in rows of grooves made with agarose gel for injection under a stereomicroscope at ×6 magnification. The needle was loaded with cells at a concentration of about 10/µL. The needle was mounted on the microinjector and approximately 4–10 nL cell suspension was injected into the yolk mass of each embryo. The same volume of the Matrigel solution or a non-tumorigenic cell suspension was injected as mock and negative controls, respectively. The injected embryos were incubated with drugs for 24 h at 28°C in fresh E3 embryo medium. At the end of the incubation, the FRET signal of zebrafish was measured on the stage of a Leica M165FC Fluorescent Stereo Microscope. The ImageJ 1.48v software (National Institutes of Health, United States) was used to quantify the FRET ratio.

### Mouse Xenograft Model

Mouse xenograft tumor was established by subcutaneous injection of reporter-labeled cancer cells. 5 × 10^6^/ml cancer cells suspended in 100 µL PBS were injected into each site of the mouse. *In vivo* imaging of the tumors in the mice was performed using a Leica microscope. Drug treatment was performed on day 7 after the inoculation of tumor cells when tumors in mice reached a size of 3–4 mm. For cisplatin treatment of Sensor C3-labelled mouse tumors, the working concentration of cisplatin was 0.6 mg/ml. Mice were injected with 6 mg/kg cisplatin after the establishment of the tumor xenograft. FRET *in vivo* was measured on the stage of a Leica fluorescence microscope. After the drug or PBS treatment, the mice were anesthetized with avertin and restrained in a specially designed holder for imaging analysis with a Leica fluorescence microscope. All animal experiments were performed following a protocol approved by the University of Macau Animal Ethics Committee (UMARE-015–2019) Animal assay.

### Statistical Analysis

All statistical data were analyzed with GraphPad Prism 8.2.1 and presented as the means ± standard error of the mean (SEM). To calculate the percentage of cell apoptosis, we measured the FRET rate (YFP/CFP rate) of tumors from three independent experiments. All data were analyzed with Student’s *t*-test, and the *p*-value < 0.05 was considered statistically significant.

## Result

### Cancer Cell Lines Expressing Sensor C3 for Detecting Apoptosis

**FIGURE 2:**
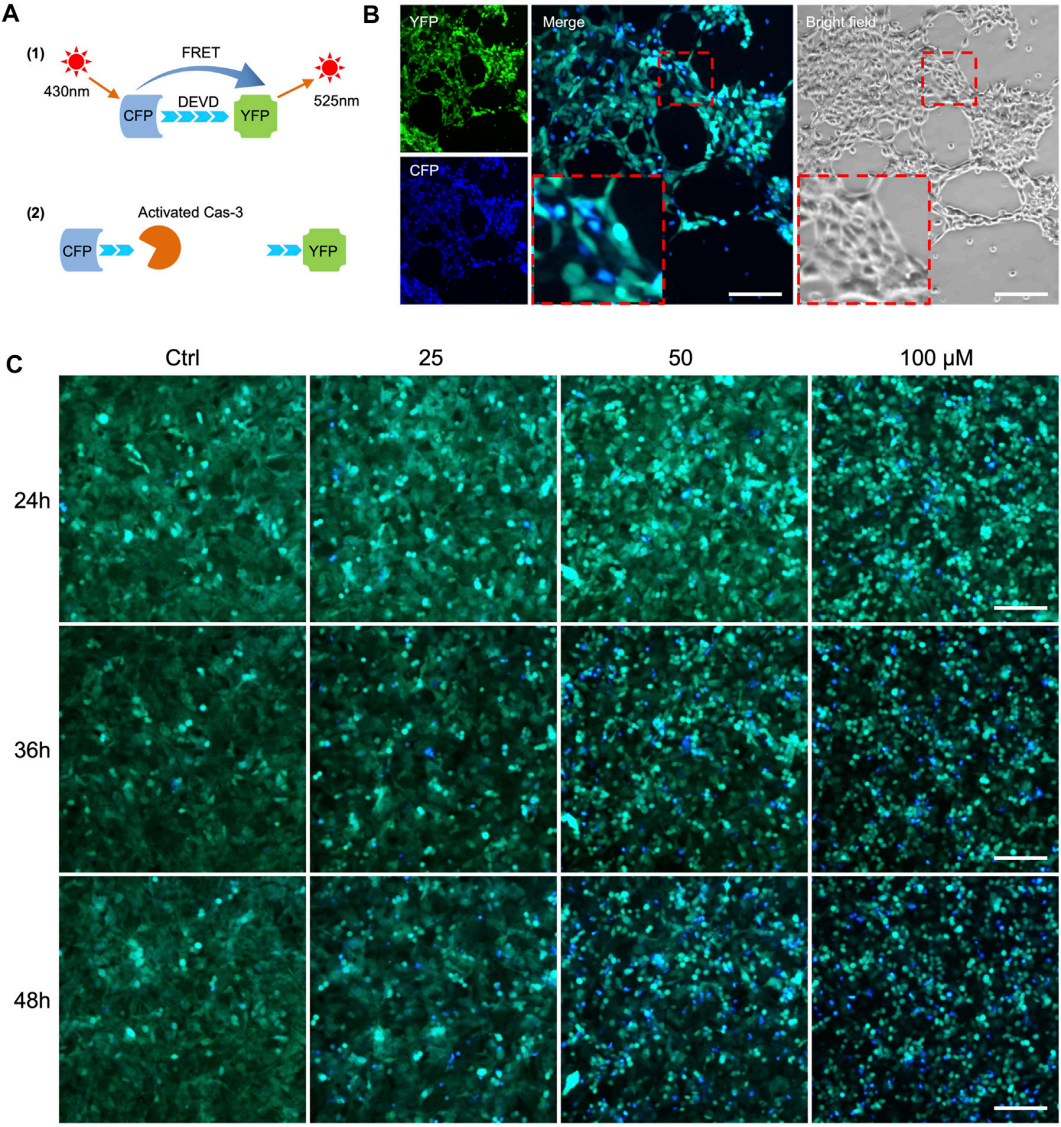
FRET-based detection of drug-induced cancer cell apoptosis *in vitro.* **(A)** Principle of caspase-3 reporter Sensor C3. **(B)** Observation of cell apoptosis in A549-C3 cells treated with 10 µM DOX for 9 h. CFP (ex: 430 nm/em: 480 nm) was merged with YFP (ex: 430 nm/em: 520 nm). Blue cells in merged images indicate apoptotic cells. **(C)** Time course and dose-dependent detection of cell apoptosis in 231-C3 cells treated with cisplatin. Blue cells in merged images indicate apoptotic cells. Scale bar = 50 µm.

To establish cellular models for detecting apoptosis *in vitro*, we introduced a previously reported sensor C3 (Regmi et al., 2018) to three cancer cell lines: MDA-MB-231 breast cancer cells, HepG2 liver cancer cells, and A549 lung cancer cells. FRET occurs when the C3-labeled cells transfer energy from CFP to YFP, causing the emission of green fluorescence (Figure 2A top). As the cells undergo apoptosis, the activated caspase-3 cut the DEVD sequence of the linker between CFP and YFP, causing less energy transfers from CFP to YFP and thus a decrease in YFP intensity. The merged FRET images in blue color can be used to report apoptosis in real-time and individual cells (Figure 2A bottom). Initially, we assessed the sensitivity of A549-C3 cells to DOX and 231-C3 to cisplatin, which are widely used drugs that kill cancer cells by inducing DNA damage and apoptosis; we found that DOX (cisplatin) induced an acute (gradual) increase of apoptotic cells (Figures 2B,C).

### Tipping Points of Apoptosis in Drug-Treated Cancer Cells

**FIGURE 3:**
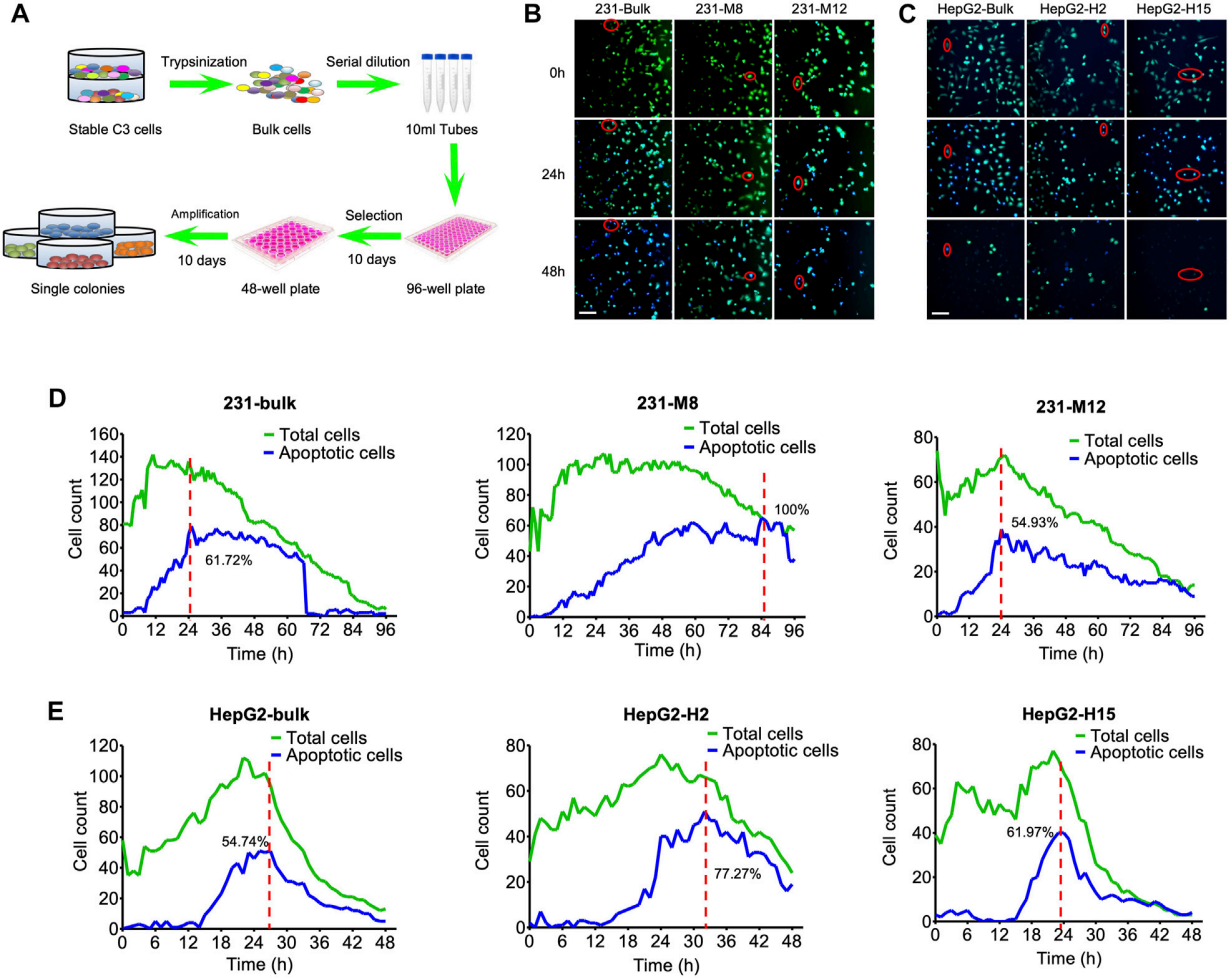
Tipping points of apoptosis in C3 cells during drug treatment. **(A)** The flow chart for the selection of single-cell colonies from bulk C3 cells. Single cell clones were harvested from bulk cells by serial dilution in 96-well plate and amplification in 48-well plate. **(B)** Images of DOX-induced cell apoptosis in single-cell colonies of MDA-MB-231-C3 cells. Red circle indicates single-cell imaging of cell apoptosis. **(C)** Images of DOX-induced cell apoptosis in single-cell colonies of HepG2-C3 cells. Red circle indicates single-cell imaging of cell apoptosis. **(D)** Time courses of DOX-induced cell apoptosis of single-cell colonies in MDA-MB-231-C3 cells. **(E)** Time courses of DOX-induced cell apoptosis of single-cell colonies in HepG2-C3 cells. Green and blue lines indicate total and apoptotic cells, respectively. Scale bar = 50 µm.

The stable C3-expressing cancer cells consisted of a mixture of transfected cells with differential C3 expression due to the differential positioning of C3 in the genome. We assumed that the heterogeneous C3 signaling at the population level does not reflect well the dynamics of caspase-3 activation at the single-cell level due to the weak fluorescence. To optimize the FRET efficiency in cancer cells for drug screening, we selected single-cell clones of C3 expression from the bulk population (Figure 3A). We then examined whether drug treatment affects the dynamics of caspase-3 activation in the single-cell clones. Both the bulk population and the single cell clones were treated with 10 µM DOX, and the corresponding cancer cell apoptosis was assessed by time-lapse photography (Figures 3B,C). For the MDA-MB-231-C3 cells, the drug response was compared between the bulk population, the M8 single clone cells, and the M12 single clone cells (Figures 3B,D). For the MDA-MB-231-C3 cells, the drug response was compared between the bulk population, the H2 single clone cells, and the H15 single clone cells (Figures 3C,E). The results revealed distinct apoptosis dynamics among the three populations, and we found that some single cell colonies were more sensitive to DOX treatment than the others.

The term tipping point describes a moment in time when a dynamical system undergoes a sudden change, shifting from one state to another (Baumuratova et al., 2013; Huang et al., 2015; Liu et al., 2016). The tipping point of apoptosis is the time when a cell shifts the cellular balance from survival to death, resulting in the initiation of its death program (Ainsworth et al., 2011; Dey et al., 2021). In the present study, the tipping points of 231-C3 cells and HepG2-C3 cells were around 6 h (Figure 3D) and 15 h (Figure 3E), respectively. For the bulk population of the 231-C3 cells (the left column of Figure 3D), the cell counts of the total cells and the apoptotic cells peak at around 10 and 24 h, respectively. For the M8 single clone of the 231-C3 cells (the middle column of Figure 3D), the cell counts of the total cells and the apoptotic cells peak at around 24 and 84 h, respectively. For the M12 single clone of the 231-C3 cells (the right column of Figure 3D), the cell counts of the total cells and the apoptotic cells peak at around 24 and 24 h, respectively. Because the peak time of M12 apoptotic cells was smaller than that of M8, we selected M12 as the representative of the MDA-MB-231 cell line for the subsequent drug screening. The HepG2 cells were similarly analyzed (Figure 3E), and H15 was selected for the subsequent drug screening.

### Drug-Induced Cell Apoptosis in Zebrafish and Mouse Xenograft Models

**FIGURE 4:**
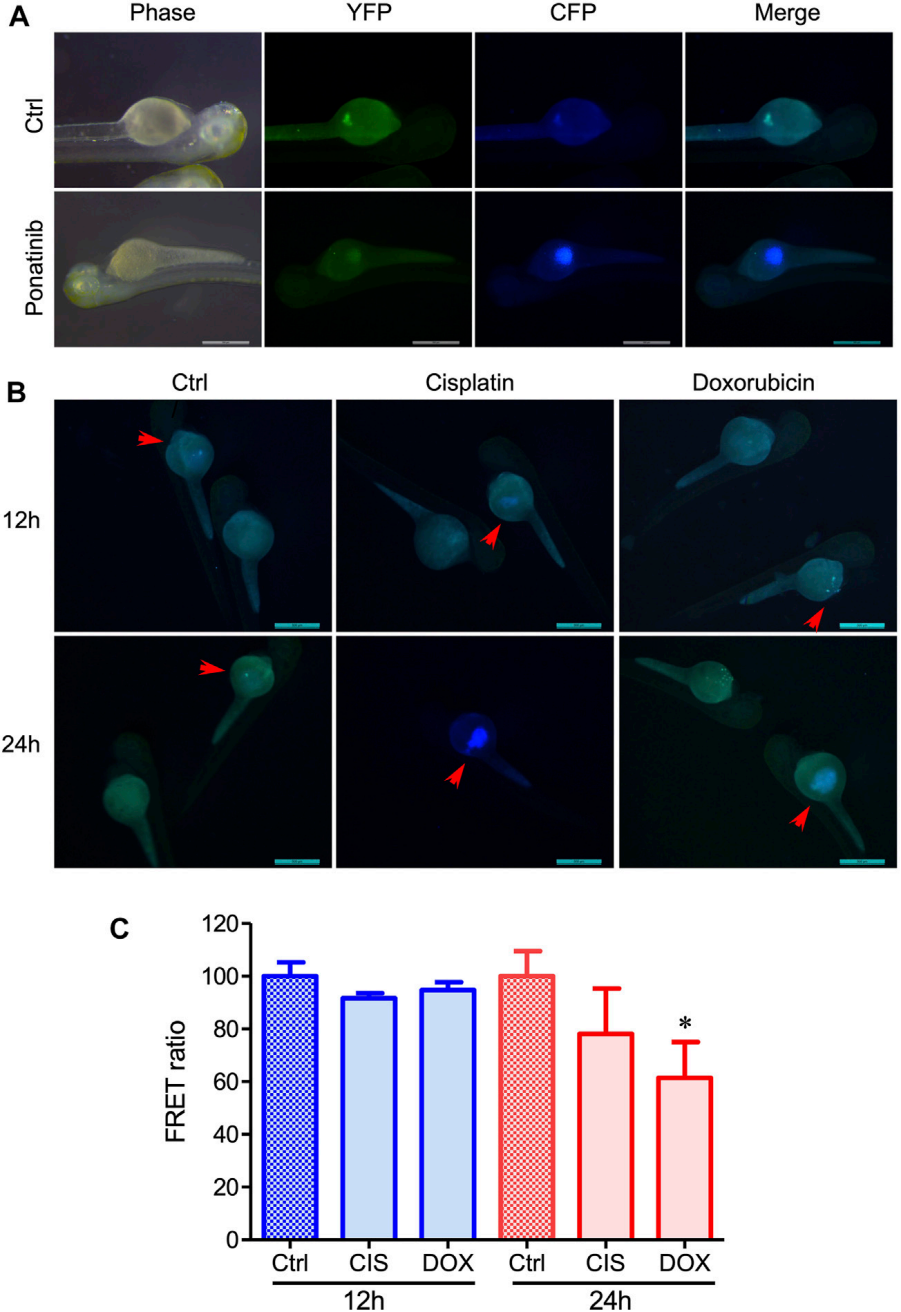
Evaluation of the in vivo drug-induced cell apoptosis in xenograft zebrafish by injecting 231-C3 cells into the zebrafish embryos. **(A)** Fluorescent images of zebrafish injected with 231-C3 cells and incubated with Ponatinib (10 μM) for 24 h. **(B)** Images of cell apoptosis in 231-C3 labeled zebrafish after 30 μM cisplatin or 2 μM DOX treatment. Arrows indicate the same zebrafishes observed at different time points. Scale bar = 500 μm. **(C)** Quantification of FRET ratio in zebrafish after 30 μM cisplatin or 2 μM DOX treatment. *n* = 6 technical replicates. Data are expressed as mean ± s.e.m. *n* = 7. *p < 0.05, by Student’s *t*-test.

To study *in-vivo* cancer cell apoptosis, we established a zebrafish cancer xenograft model and performed a time-lapse experiment monitoring FRET changes after drug treatment. After injection of 231-C3 cells (100–150) into the yolk of 2-dpf zebrafish embryos, the C3-labeled cells were observed in the recipient embryos, along with their development (Figure 4). The zebrafish embryos were then treated with 10 μM ponatinib, which induced cell apoptosis (Figure 4A). The C3 expressing cancer cells in zebrafish embryos showed a reduction of FRET signal (blue color in the images), demonstrating the successful induction of apoptosis at 24 h post treatment. We also tested cisplatin and DOX, which both have been proved to be effective in inducing cancer cell apoptosis (Figures 4B,C). These experiments demonstrate the potential of FRET technique in the monitoring of the drug effectiveness and *in vivo* pharmacodynamics based on C3 xenograft zebrafish models.

**FIGURE 5:**
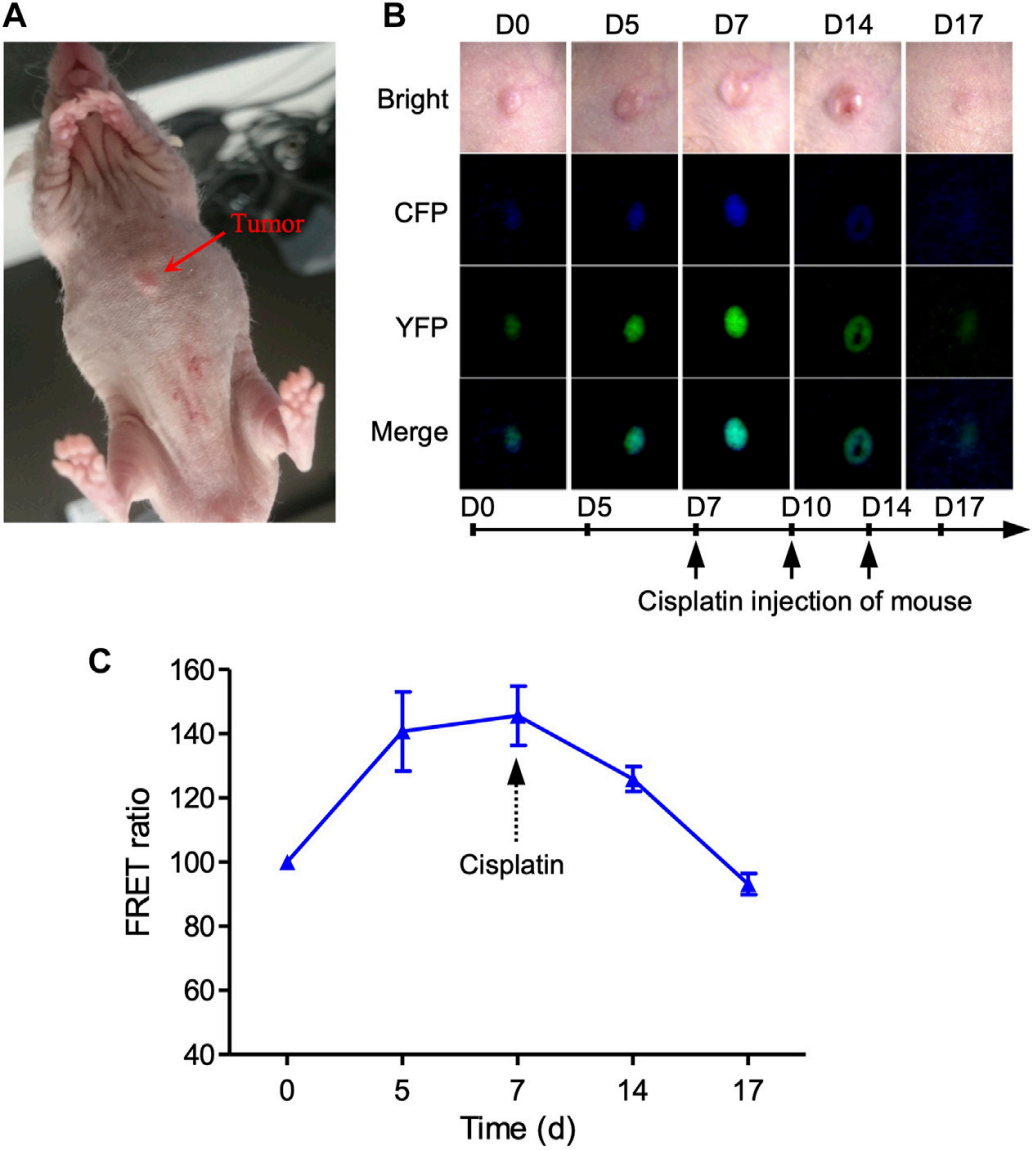
Imaging of cell apoptosis in xenograft tumor mouse *in vivo*. **(A)** Imaging of 231-C3 xenograft nude mice and FRET channels of a tumor with no drug treatment. 100 µL 5×10^6^/ml 231-C3 cells were injected into each site of nude mice. Mice were injected with 6 mg/kg cisplatin after the establishment of the tumor xenograft. **(B)** Imaging of 231-C3 xenograft tumor nude mice with cisplatin treatment. **(C)** Quantification of FRET ratio in xenograft nude mice tumors after 6 mg/kg cisplatin treatment. *In vivo* imaging of mouse tumor was performed using a Leica fluorescent microscope. CFP (ex: 440 nm; em: 480 nm), YFP (ex: 440 nm; em: 535 nm). Data are expressed as mean ± s.e.m. *n* = 3.

We also created a xenograft mouse model by injecting MDA-MB231-C3 cells subcutaneously into mice, allowing us to track the C3 cells’ FRET changes in *vivo* mouse model (Figure 5A). The tumor grows over time in nude mice before cisplatin treatment, accompanied by an increase in the fluorescence intensity of CFP and YFP (Figure 5B). Seven days after the cisplatin treatment, nude mice showed continuous tumor regression, as well as the decrease of FRET ratio and the intensity of CFP/YFP (Figures 5B,C), demonstrating that cisplatin can inhibit tumor growth by inducing cell apoptosis in the nude mice. However, there was a slight decrease in the FRET ratio compared to day 0 group due to the skin of the mice (Figure 5C). Overall, these results suggested that FRET technique has great potential to explore the pharmacodynamics of anti-cancer drugs based on C3 xenograft mouse models with an improved fluorescence microscope in terms of high depth resolution transmission.

## Discussion

Traditionally, the evaluation of drug responses relies on fixation and dye labeling that are destructive and toxic to cells (Foglieni et al., 2001; Johnson et al., 2013; Kilgore et al., 2013), with valuable cytomic and metabolic information discarded as a consequence. Due to the lack of effective predictive biomarkers or reporters for drug discovery *in vivo*, it takes a long time to translate cell-based drug screening to animal models. To address this issue, we established cancer cell lines that express FRET-based C3, which acts as a reporter to screen anti-cancer drugs, and then injected the established cancer cells into zebrafish and mouse to develop the corresponding xenograft models for the efficient drug screening *in vivo*. The xenograft models have been well established because the drugs were effective in the induction of apoptosis and the inhibition of tumor growth.

Since the stable cells expressing C3 had a heterogeneous genetic background and showed distinct phenotypes (Figure 3), single cells from the bulk population were selected and screened, and MDA-MB-231-C3-S12 and HepG2-C3-H15 were used as sensitive clones for drug screening. We found that the chemotherapeutic drug DOX induced apoptosis in MDA-MB-231-C3-S12 and HepG2-C3-H15 cells. We then established FRET-based caspase reporters capable of detecting caspase-3 activation and used them to evaluate the drugs *via* the established zebrafish and mouse xenograft models (Figures 4, 5). However, approximately 50% of zebrafish died after the injection of cancer cells, implying that zebrafish has limitations as a model for evaluating the ability of drugs to induce apoptosis. In the mouse xenograft model, the FRET ratio decreased slightly after drug treatment, that might be because of the fluorescence of YFP and CFP is too weak to be detected *in vivo* (Dye, 2005; Mc Intyre et al., 2007) or the thick mouse skin and the underlying tissues could interfere with the CFP, YFP, and apoptotic fluorescent, rendering our equipment unable to detect FRET changes. Thus, further investigations of these models with improved conditions are needed (Hirata and Kiyokawa, 2016).

Our C3-based platform is more powerful and reliable than the luciferase-based method for observing cell apoptosis *via* fluorescence microscope. The luciferase-based method needs a substrate called luciferin (Hickson et al., 2010) whereas the FRET-based C3 platform is substrate-free. The luciferase-based imaging often causes severe photobleaching, which is not a problem of the FRET-based C3 platform. Therefore, our platform and approach should be suitable for large-scale drug screening *in vitro* to narrow down potential candidates for the further analysis *in vivo*. The development of FRET-based anti-cancer drug screening, from *in vitro* to *in vivo*, has provided a feasible *in vivo* approach to assess the efficacy of novel drug candidates in inducing cancer cell apoptosis. Thus, this approach has the potential to be used in preclinical experiments to provide personalized therapeutic options for individual patients.

## Data Availability

The raw data supporting the conclusion of this article will be made available by the authors, without undue reservation.
