# Corrigendum: An *in vivo* fluorescence resonance energy transfer-based imaging platform for targeted drug discovery and cancer therapy

**DOI:** 10.3389/fbioe.2022.1039177

**Published:** 2022-09-26

**Authors:** Fuqiang Xing, Nana Ai, Shigao Huang, Cheng Jiang, Muhammad Jameel Mughal, Wei Ge, Guanyu Wang, Chu-Xia Deng

**Affiliations:** ^1^ Cancer Center, Faculty of Health Sciences, University of Macau, Macau SAR, China; ^2^ Department of Biology, School of Life Sciences, Guangdong Provincial Key Laboratory of Computational Science and Material Design, Southern University of Science and Technology, Shenzhen, China; ^3^ Guangdong Provincial Key Laboratory of Biomedical Imaging and Guangdong Provincial Engineering Research Center of Molecular Imaging, The Fifth Affiliated Hospital, Sun Yat-sen University, Zhuhai, China; ^4^ Centre of Reproduction, Development and Aging, Faculty of Health Sciences, University of Macau, Macau SAR, China; ^5^ School of Life and Health Sciences, The Chinese University of Hong Kong, Shenzhen, China

**Keywords:** apoptosis, cancer therapy, drug discovery, FRET technique, xenograft model

In the published article, there was an error in the author list. The authors Nana Ai, Wei Ge, and Chu-Xia Deng were erroneously excluded. The corrected author list appears below.

“Fuqiang Xing^1,2,3^, Nana Ai^4^, Shigao Huang^1^, Cheng Jiang^5^, Muhammad Jameel Mughal^1^, Wei Ge^4^, Guanyu Wang^2,5^* and Chu-Xia Deng^1^*”.

The affiliations list, author contributions, and acknowledgements statements were updated to reflect the addition of these authors.

